# Elastography of the Intestinal Mucosa of Dogs With Chronic Inflammatory Enteropathy

**DOI:** 10.1155/vmi/5562334

**Published:** 2026-07-29

**Authors:** Iago Martins Oliveira, Rafaela Rodrigues Ribeiro, Maria Eduarda Cardoso Cysneiros, Vanessa Rezende Moraes, Lucas Rodrigues Ferreira, Wanessa Patrícia Rodrigues Da Silva, Murilo Rodrigues De Souza, Rafael Antônio Lopes Xavier, Marco Augusto Machado Silva, Naida Cristina Borges

**Affiliations:** ^1^ School of Veterinary Medicine and Animal Science, Universidade Federal de Goiás, Rodovia Goiânia-Nova Veneza, km 8 Campus Samambaia, Goiânia 74690-900, Goiás, Brazil, ufg.br; ^2^ Pontifical Catholic University of Goiás, Campus II Av. Engler s/n-Jardim Mariliza, Goiânia Goiás, 74885-460, Brazil, pucgoias.edu.br

**Keywords:** canine, fibrosis, hardness, intestinal elasticity

## Abstract

The aim of this study was to determine the association between intestinal elastography and laboratory, clinical, ultrasound, endoscopic, and histopathological parameters in dogs with chronic inflammatory enteropathy (CIE). Twelve dogs were assessed and divided into two groups: six dogs with CIE (EG) and six healthy dogs (CG). Strain ratio (SR) values for the duodenum in the EG ranged from 1.01 to 3.3, with a mean of 2.105 ± 1.016, while in the CG, they ranged from 0.2 to 0.96, with a mean of 0.5667 ± 0.3133. For the jejunum, the values in the EG ranged from 1.19 to 2.76, with an average of 1.867 ± 0.5711, and in the CG, they ranged from 0.15 to 0.72, with an average of 0.4667 ± 0.2439. The results showed that the EG dogs had higher values in the elastography parameters than the CG, with significant differences in the SR of the duodenum (*p* = 0.0053) and jejunum (*p* = 0.0003). There was no significant correlation between intestinal elasticity and the variables weight and age of the dogs, either in the EG or in the CG. For the clinical, laboratory, and ultrasound parameters, there were weak negative and positive correlations between the variables analyzed, except for a strong, negative, and statistically significant correlation (*r* = −0.886, *p* = 0.033) between jejunal SR and serum albumin. Digestive endoscopy and histopathological analysis showed varied lesions, with no significant correlation with elastography values. It is concluded that strain elastography is a viable technique for assessing the intestines of dogs with CIE, showing higher intestinal stiffness values compared to healthy dogs. It was not possible to infer that increased SR suggests intestinal fibrosis and that mucosal hardness is associated with greater clinical alterations and complementary tests. Despite this, we suggest that the results found be applied in further studies with a larger number of animals, measuring more specific biological markers for the canine gastrointestinal tract and making better use of the samples obtained from intestinal biopsy and histopathology.

## 1. Introduction

Canine chronic inflammatory enteropathy (CIE) is diagnosed based on the clinical manifestation of vomiting, diarrhea, changes in appetite, and weight gain, lasting more than 3 weeks, evidence of inflammatory infiltrate in the intestinal mucosa and differential diagnosis of systemic diseases. After excluding infectious, neoplastic, metabolic, renal, and hepatic causes, intestinal disease can be classified according to the response to therapy. Monitoring, assessing the progression, and staging of the inflammatory process of this disease in dogs are challenging due to the heterogeneity of pathogenesis [1, 2]. Because of this, new diagnostic options are constantly being developed and studied [3].

Thus, elastography can help monitor inflammation as it is an ultrasound (USG) technique that assesses the elasticity of structures and is an innovative way of studying tissue mechanical properties [4]. The modality has been used to analyze various organs in animals [5], and for intestinal evaluation, this category of imaging exam has emerged as a way of evaluating the physiological elasticity of the intestinal mucosa in dogs; however, there is still no application of the exam in animals with enteropathies [6]. In humans, elastography has already proved useful in assessing intestinal diseases, with its potential use in stenoses, ulcerations, fistulas [7, 8], and in mapping fibrosis resulting from Crohn’s disease [9].

Intestinal USG evaluation provides relevant information on topography, wall thickening, layer stratification, echogenicity, motility, luminal content, and characteristics of adjacent structures such as lymph nodes, peritoneum, and other structures attached to the gastrointestinal tract. In this way, the examination can be indicated for animals with intestinal clinical signs [10]. Thickening, loss of parietal stratification, increases or decreases in echogenicity, and hyperechogenic striations in the mucosa are findings described on USG in dogs with intestinal disorders [11].

In addition to imaging modalities, laboratory markers can be used in the diagnosis, therapeutic monitoring, and prognosis of dogs with chronic enteropathy [12]. However, due to the variability of the diseases that affect the intestines of dogs, it has been found that there is a need to standardize more specific laboratory markers for gastrointestinal assessment in this species [13]. Based on this, it is important to apply a biological indicator to help assess the functionality of the organ, to recognize the risk of developing the disease, and to have diagnostic value [14]. Therefore, the biomarkers used in canine intestinal diseases can be classified as functional, biochemical, inflammatory, cellular, genomic, proteomic, metabolomic, and microbiological [12].

More specifically, digestive endoscopy allows for direct, noninvasive assessment of the intestinal lumen, and endoscopic intestinal biopsy provides materials for histopathological analysis [15]. Histological evidence of mucosal inflammation after clinical, dietary, and laboratory screening confirms the diagnosis of CIE, but does not classify it [16]. For histological description, guidelines were created that included visual description and grading of morphological and inflammatory changes in the intestines [17].

Our hypothesis is that the strain ratio (SR) determined by strain elastography is increased in dogs with CIE, indicating greater intestinal rigidity, and, therefore, that the test is a potential early and noninvasive method of detecting fibrosis. In addition, we expect increased intestinal hardness to be related to more severe clinical activity index scores, greater USG thickening, and more evident alterations in laboratory, macroscopic, and microscopic evaluation of the mucosa. The aim of this study was therefore to evaluate the intestines of dogs with CIE, to define the deformation rates of these animals, and to correlate intestinal elasticity values with clinical, laboratory, USG, endoscopic, and histopathological parameters.

## 2. Materials and Methods

### 2.1. Animals

The study was approved by the Ethics Committee for the Use of Animals at the Federal University of Goiás (UFG) under protocol numbers 071/20 and 041/24. The experiment was carried out at the Veterinary Hospital of the UFG Veterinary and Zootechnical School and at veterinary clinics in the city of Goiânia. The animals used were canines with chronic gastrointestinal signs lasting more than 3 weeks and healthy dogs with no history of previous illnesses in the last 3 months. All dogs underwent anamnesis and a complete physical examination, including assessment of consciousness, hydration status, mucous membrane color, capillary refill time, rectal temperature, pulse rate, palpable lymph nodes, and cardiopulmonary auscultation. A specific gastrointestinal examination was also performed, including evaluation of the oral cavity, inspection and palpation of the esophageal region, and abdominal inspection, palpation, and percussion. The owners authorized the use of their animals by signing an informed consent form.

### 2.2. Experimental Design

Given the confirmation of chronicity in symptomatic animals, screening was carried out using laboratory tests, USG, dietary changes, and the use of antiparasitic drugs. Therefore, a blood count and the following serum biochemical tests were carried out: creatinine, alanine aminotransferase (ALT), alkaline phosphatase (ALP), albumin, and total cholesterol, as well as basal cortisol, canine trypsinogen, basal bile acids, cobalamin, folate, and C‐reactive protein (CRP); stool parasitology using the direct method and in a saturated zinc solution with a single fecal sample; and an immunochromatographic test to detect anti‐*Giardia duodenalis* antigens (Conclue Giardíase, Ourofino, Vinhedo, São Paulo, Brazil). A total abdominal USG was performed, and a hypoallergenic diet with hydrolyzed protein was prescribed for 30 consecutive days in a restrictive manner. All the animals received fenbendazole (Fenzol Pet, Agener União, São Paulo, Brazil) 50 mg/kg orally, once a day, for 3 days, and after 15 days, the protocol was carried out again.

Fifteen male and female dogs aged between 12 months and 10 years and weighing between 1 and 30 kg were evaluated. The inclusion criteria were the presence of chronic gastrointestinal clinical signs and no response to screening. Exclusion criteria were animals that showed complete improvement of clinical signs with a change in diet, as well as those in which alterations not associated with the gastrointestinal tract were found in laboratory and USG tests. Therefore, eight animals were excluded because they showed clinical remission with the dietary change and one dog was excluded because it had exocrine pancreatic insufficiency (EPI).

Therefore, a group was formed consisting of six dogs with persistent signs that were not conclusive in the screening, called the enteropathy group (EG). Subsequently, these animals were referred for upper digestive endoscopy and histopathological evaluation of the duodenum, which concluded that all the individuals had CIE. In addition, semiquantitative elastography of the mucosa of the duodenum and jejunum was also carried out.

In the asymptomatic animals, after a general and specific clinical assessment, a blood count, ALT, ALP, albumin, creatinine, total cholesterol, and USG were carried out. The following inclusion criteria were used to form the experimental group: no clinical signs, normal physical examination, and laboratory and USG parameters. Exclusion criteria were the presence of diseases and alterations in the complementary tests carried out. Eight male and female dogs aged between 12 months and 8 years and weighing between 1 and 25 kg were assessed. Of these, two animals were excluded because they had laboratory alterations and so another experimental group was formed, called the control group (CG) containing six healthy dogs. Intestinal elastography was then carried out using the same methodology as for the EG.

### 2.3. Clinical Activity of the Disease

The EG dogs showed gastrointestinal clinical manifestations (≥ 3 weeks duration) intermittently. Signs included vomiting, diarrhea, nausea, flatulence, abdominal pain, and weight loss. These occurred in isolation or in association. The severity of the enteropathy was graded using a clinical activity index, the Canine Chronic Enteropathy Activity Index (CCECAI) [18].

The index considers behavior, appetite, vomiting frequency, fecal consistency, defecation frequency, weight loss, serum albumin, presence of ascites, and peripheral edema and pruritus as parameters. The criteria are evaluated on a scale of 0–3, and the resulting CCECAI score was formed by summing the data. The total index score can range from 0 to 27, and based on this score, chronic bowel disease is classified as clinically insignificant (0–3 points), mild (4–5 points), moderate (6–8 points), severe (9–11 points), or very severe (score ≥ 12).

### 2.4. Serum Biomarkers

For blood collection, the animals were fasted for 12 h, and all analyses were carried out within 2 h of collection. Five milliliters of blood was collected by puncturing the external jugular vein. The blood was placed in a tube containing 10% disodium ethylenediaminetetraacetic acid (EDTA) and in tubes without anticoagulant. The blood count was done using an automatic analyzer (Celltac *α* MEK 6550, Nihon Kohden, Japan) to obtain the parameters of total leukocyte count, red blood cell count, hemoglobin, mean corpuscular volume (MCV), mean corpuscular hemoglobin concentration (MCHC), and total platelet count (PLT). Hematocrit and total plasma proteins (TPP) were analyzed manually using the microhematocrit technique and refractometry, respectively. Blood smears were prepared immediately after collection and stained with Romanowsky’s stain (Rapid Panoptic, Laborclin, Pinhais, Paraná, Brazil) for morphological analysis of the cells, including the differential count of leukocytes by light microscopy.

The blood that was separated into tubes without anticoagulant was centrifuged for 5 minutes at a speed of 3600 revolutions per minute to obtain the blood serum. In the serum biochemistry evaluation, ALT, creatinine, ALP, total cholesterol, and albumin concentrations were measured. All analyses were carried out on fresh samples using an automated biochemistry device (CM 250, Wiener, Argentina). The values obtained in this analysis were dosed using commercial reagent kits (Biotécnica and Doles).

During the screening period of the EG animals, tests were carried out to help differentiate nonintestinal enteropathies, such as basal bile acids by the cyclic enzymatic method to rule out portosystemic shunting, basal cortisol to screen for atypical hypoadrenocorticism, and canine trypsinogen to differentiate EPI. In addition, cobalamin and folate were measured as intestinal absorption markers by electrochemiluminescence and CRP by immunochromatography as an inflammatory marker. These markers were not carried out on the CG dogs as there was no evidence of systemic or intestinal disease in these animals.

### 2.5. USG

The USG examination was carried out on the dogs in both groups, and the animals were fasted for 12 h on food and 6 h on water. SAEVO FT422 equipment was used, coupled to a microconvex and linear transducer set to frequencies of 5.0–8.0 MHz, depending on the physical size of the dog being examined. The evaluations were carried out by the same examiner. The dogs were placed in dorsal decubitus, and, before the evaluation, the abdomen was thoroughly trichotomized and acoustic gel was applied to ensure better contact with the skin.

The size, shape, contour, echogenicity, and echotexture of the abdominal organs were analyzed. The examination approach followed a ventrolateral sequence from left to right, including assessment of the urinary vesicle, left kidney, left adrenal gland, spleen, stomach, liver and gallbladder, right kidney, right adrenal gland, duodenum, pancreas, jejunum, ileum, and colon. The abdominal lymph nodes were also assessed. In males, the prostate and testicles were examined, and in females, the right and left ovaries and the uterus were examined. In addition, the echogenicity of the left kidney was compared with that of the spleen, while the echogenicity of the right kidney was compared with that of the liver.

The USG of the intestinal segments followed the recommendations and reference values described in the literature [19]. The aim was to identify the parietal stratification and measure in centimeters, visualizing the five layers of the intestinal wall. The total diameters of the intestinal walls were measured using the cursor in the USG machine software.

### 2.6. Semiquantitative Strain Elastography

Elastography was carried out on the EG and CG dogs with the same fasting recommendations as for the USG examination. The technique was based on the semiquantitative deformation method using a SAEVO FT422 device and a linear transducer with a frequency of 7.5–10 MHz (based on the animal’s physical size). All the evaluations were carried out by the same examiner with proficiency in the area (Silva, WPR). During the examinations, the transducer was positioned perpendicular to the animals’ abdomens, without lateralization, followed by two cycles of compression and decompression. The elastographic map was generated on a colorimetric scale, overlaying the image in the B‐mode, ranging from shades of blue to red, with blue indicating maximum stiffness, green for intermediate stiffness, and red for maximum elasticity. The images were acquired with uniform color filling in the structures observed, especially in the intestinal mucosa, allowing for visual scanning and calculations of semiquantitative data.

The mucosa of the duodenum and proximal jejunum was evaluated in cross section. The image of the intestines was projected twice, displaying both the B‐mode USG and the elastography, to maintain the scanning plane during imaging. In addition, the scan generated a waveform indicator, called the elastographic tension curve, which represented the compression of the transducer during the scan.

In addition to visual observation, the mucosa of the duodenum and proximal jejunum was evaluated by relative hardness measurement, using a cursor in the device’s software in the region of interest (ROI) for elastography. This allowed a measurement to be formed based on the deformation calculations obtained using a selected ROI in the intestinal mucosa and another in the adjacent mesentery. The ROIs were drawn in a similar way, with a circular shape, at the same depth and in the horizontal direction of the cross section of the intestinal segments evaluated. The SR was calculated from the ratio of the ROIs.

### 2.7. Upper Digestive Endoscopy

For the endoscopic procedure, the animals in the EG underwent food and water fasting. Endoscopies were performed by the same examiner on all the animals in this study, as was the classification of endoscopic parameters. The equipment used was a model EG‐250PE5 gastroscope (Fujinon EPX‐2200) with a total length of 140 cm, a working length of 110 cm, a diameter of 8.1 mm, a 2.8‐mm suction and biopsy channel, coupled with a surgical aspirator (Aspira Max NS Industrial, São Paulo, SP), insufflator and 250‐W halogen light source (Ferrari Medical, São Paulo, SP), and fenestrated, oval, short 2.3‐mm biopsy forceps with needle (Ferrari Medical, São Paulo, SP). Under general anesthesia, the dogs were positioned in the left lateral decubitus position with the head and cervical region distended and the oral cavity kept open.

The endoscopic parameters for assessing the severity of the lesions observed during the examination followed a four‐point grading system [20], with 0—compatible with normal mucosa or absence of detectable lesions, 1—mild lesions, 2—moderate lesions, and 3—severe lesions. The scores were only performed during duodenoscopy, and the criteria considered were hyperemia, edema, friability, hemorrhage, erosion/ulceration and lacteal dilation. After the endoscopic indices of the severity of the enteropathy had been carried out, the material was collected.

The fragments were collected at the end of the examination to prevent the hemorrhage resulting from the biopsies from compromising the evaluation of the mucosa. Fifteen fragments were collected from the duodenum. In this procedure, the forceps were introduced through the working channel of the endoscope and exposed in the duodenum and then in the stomach. Once open, the forceps were pressed against the duodenal wall and then closed to seize a fragment of the mucosa. The clamp with the sample was then removed and passed back through the biopsy channel until it was exteriorized. Using a 25 × 0.70‐mm hypodermic needle, the fragment was removed from the biopsy forceps, distended, and covered in absorbent paper before being immersed in a vial containing 10% buffered formalin. After the biopsies were taken, the insufflated air was aspirated, and the equipment was removed. At the end of all the procedures, the equipment used was cleaned with enzymatic detergent (Ecosime Lenzafarm, Belo Horizonte‐MG) and disinfected with 2% glutaraldehyde (Glutaron, Rio Química, São José do Rio Preto‐SP) for 30 min.

### 2.8. Histopathology

All the samples were processed and evaluated by the same pathologist, as well as determining the frequency and classification of the alterations seen in the histopathological examination. The histological specimens were dehydrated, diaphanized, and embedded in paraffin. The sections were made using a rotary microtome, followed by distension on frosted glass slides, stained with hematoxylin and eosin (HE), covered in synthetic resin, and evaluated under an optical microscope at 40x magnification.

The histological parameters assessed in the duodenum were classified in scores from 1 to 3, according to the severity of the lesions, following the guidelines standardized in the literature [21]. Therefore, Grade 0 refers to absent lesions, 1 to discrete lesions, 2 to moderate lesions, and 3 to severe lesions. In addition, the classification system includes the representativeness of the samples analyzed, which are categorized as adequate, inadequate, and marginal. The parameters assessed included morphological and inflammatory patterns. The morphological criteria included damage to the surface epithelium, shortening of villi, distortion of crypts, dilation of milk vessels, and mucosal fibrosis. To assess inflammation, we considered the presence of intraepithelial lymphocytes, lymphocytes, eosinophils, neutrophils, and macrophages in the lamina propria.

### 2.9. Statistical Analysis

The data obtained were presented using descriptive statistics. The Shapiro–Wilk test was used to establish normality. Unpaired and parametric data were submitted to the *t*‐test with Welch’s correction. In all analyses, differences were considered significant when *p* < 0.05.

Pearson’s correlation test was used to assess the relationship between intestinal SR and the weight and age of the animals in the EG and CG. Spearman’s correlation test was used to evaluate the relationship between the intestinal SR of the EG dogs and the variables red erythrocytes, PLT, total leukocytes, TPP, albumin, total cholesterol, CRP, cobalamin, folate, CCECAI, USG measurements, and endoscopic and histopathological scores. For interpretation, 0.00–0.19 was considered a very weak correlation, 0.2–0.39 a weak correlation, 0.4–0.69 a moderate correlation, 0.7–0.89 a strong correlation, and above 0.90 a very strong correlation. These reference values were considered when interpreting all the correlation tests in this study. The data were evaluated, and the graphs were generated using GraphPad Prism 10.

## 3. Results

The animals in the EG were of different breeds, comprising 33.3% French Bulldogs (*n* = 2/6), 33.3% Shih Tzu (*n* = 2/6), 16.6% Border Collie (*n* = 1/6), and 16.6% Poodle (*n* = 1/6). With regard to sex, 100% (*n* = 6/6) of the animals were castrated females. The average weight was 8.083 ± 2.516 kg, and the average age was 3.417 ± 2.498 years. In the CG, 100% (*n* = 6/6) of the animals had no defined breed, 83.3% (*n* = 5/6) were males, and 16.6% (*n* = 1/6) were castrated females. The average weight was 16.87 ± 4.143 kg, and the average age was 9.0 ± 3.742 years.

Weak, negative relationships were observed between duodenal SR and weight (*r* = −0.240, *p* = 0.647) and age (*r* = −0.034, *p* = 0.949). In the analysis of jejunal SR, there was also a weak positive correlation with weight (*r* = 0.490, *p* = 0.324) and a negative correlation with age (*r* = −0.328, *p* = 0.526). Similar results were obtained in the CG, where the association with the SR of the duodenum showed a weak positive correlation with weight (*r* = 0.448, *p* = 0.373) and a negative correlation with age (*r* = −0.601, *p* = 0.207) and in the jejunum, weak negative correlations with weight (*r* = −0.626, *p* = 0.184) and age (*r* = −0.277, *p* = 0.595).

As for laboratory parameters, the following mean values and standard deviations were observed in the CG dogs: erythrocytes 7.768 ± 0.5333 106/μL, platelets 223.2 ± 22.74 103/μL, total leukocytes 10,417 ± 3347 μL, TPP 7.850 ± 1.340 mg/dL, albumin 3.228 ± 0.5328 g/dL, and total cholesterol 236.0 ± 53.11 mg/dL. In the EG, there were erythrocytes 7.145 ± 0.6793 106/μL, platelets 331.5 ± 82.44 103/μL, total leukocytes 14,820 ± 7306 μL, TPP 7.267 ± 0.3983 mg/dL, albumin 3.292 ± 0.3711 g/dL, total cholesterol 176.8 ± 48.99 mg/dL, CRP 17.15 ± 11.49 mg/L, cobalamin 878.5 ± 661.7 pg/mL, and folate 9.280 ± 1.939 ng/mL. The descriptive statistics for the results of both groups are shown in Table 1.

**TABLE 1:** Mean, standard deviation, mean error, mean confidence interval (CI), and coefficient of variation of the variables ROI 1 and ROI 2 of the duodenum, ROI 1 and ROI 2 of the jejunum, strain ratio (SR) of the duodenum and jejunum, weight, age, and laboratory parameters of the animals in the EG and CG, respectively.

Variable	Mean	Standard deviation	Mean error	Mean CI (95%) low	Mean CI (95%) high	Coefficient of variation (%)
*Enteropathy group (EG)*						
ROI 1 duodenum	0.9133%	0.3665	0.1496	0.5287	1.298	40.13
ROI 2 duodenum	0.4833%	0.1935	0.07898	0.2803	0.6864	40.03
SR duodenum	2.105	1.016	0.4148	1.039	3.171	48.26
ROI 1 jejunum	1.048%	0.5367	0.2191	0.4851	1.612	51.20
ROI 2 jejunum	0.5900%	0.3513	0.1434	0.2214	0.9586	59.54
SR jejunum	1.867	0.5711	0.2332	1.267	2.466	30.60
Weight	8.083 kg	2.516	1.027	5.443	10.72	31.12
Age	3.417 years	2.498	1.020	0.7948	6.039	73.12
Erythrocytes	7.145 × 10^6^ μL	0.6793	0.2773	6.432	7.858	9.508
Platelets	331.5 × 10^3^/μL	82.44	33.66	245.0	418.0	24.87
Total leukocytes	14,820/μL	7306	2983	7150	22,480	49.31
TPP	7.267 g/dL	0.3983	0.1626	6.849	7.685	5.482
Albumin	3.292 g/dL	0.3711	0.1515	2.902	3.681	11.27
Total cholesterol	176.8 mg/dL	48.99	20.00	125.4	228.2	27.71

*Control group (CG)*						
ROI 1 duodenum	0.4833%	0.2926	0.1194	0.1763	0.7903	60.53
ROI 2 duodenum	1.000%	0.5071	0.2070	0.4679	1.532	50.71
SR duodenum	0.5667	0.3133	0.1279	0.2379	0.8954	55.29
ROI 1 jejunum	0.3550%	0.1823	0.07442	0.1637	0.5463	51.35
ROI 2 jejunum	1.107%	0.8508	0.3473	0.2139	1.999	76.88
SR jejunum	0.4667	0.2439	0.09959	0.2107	0.7227	52.27
Weight	16.87 kg	4.143	1.691	12.52	21.21	24.56
Age	9.0 years	3.742	1.528	5.073	12.93	41.57
Erythrocytes	7.768 × 10^6^/μL	0.5333	0.2177	7.209	8.328	6.865
Platelets	223.2 × 10^3^/μL	22.74	9.282	199.3	247.0	10.19
Total leukocytes	10,417/μL	3347	1366	6904	13,929	32.13
TPP	7.850 g/dL	1.340	0.5470	6.444	9.256	17.07
Albumin	3.228 g/dL	0.5328	0.2175	2.669	3.787	16.50
Total cholesterol	236.0 mg/dL	53.11	21.68	180.3	291.7	22.51

**FIGURE 1:**
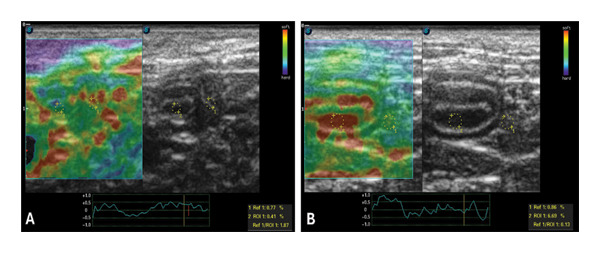
Elastography of jejunal mucosal deformation in a dog with chronic inflammatory enteropathy and in a healthy dog. The color elastogram and B‐mode ultrasound image of the jejunum in cross section. ROI 1 can be seen in the mesenteric region and ROI 2 in the dorsal mucosa of the jejunum. (A) A dog with chronic inflammatory enteropathy in which the visual distribution of colors is represented by shades of blue and green in the mucosa, with a strain ratio of 1.87. (B) A healthy dog in which the visual distribution of colors is represented by shades of red and green in the mucosa, with a strain ratio of 0.13.

The results of the elastography showed that in the EG animals, ROI 1 in the mesentery adjacent to the duodenum averaged 0.9133 ± 0.3665% and ROI 2 in the duodenal mucosa averaged 0.4833 ± 0.1935%, and ROI 1 in the mesentery adjacent to the jejunum averaged 1.048 ± 0.5367% and ROI 2 in the jejunal mucosa averaged 0.5900 ± 0.3513%. In the CG, the mean value of ROI 1 found in the mesentery adjacent to the duodenum was 0.4833 ± 0.2926%, that of ROI 2 in the duodenal mucosa was 1.000 ± 0.5071%, that of ROI 1 of the mesentery adjacent to the jejunum was 0.3550 ± 0.1823%, and that of ROI 2 in the jejunal mucosa was 1.107 ± 0.8508%. The elastographic map that was generated between the groups of dogs in the study and the markings of the ROIs in the intestinal mucosa and mesentery are shown in Figure 1.

**FIGURE 2:**
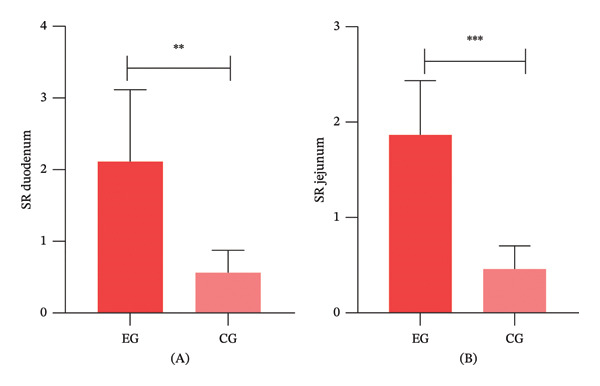
Mean values and standard deviation of the strain ratio (SR) of dogs in the enteropathy group (EG) and control group (CG). (A) Comparison between duodenum SRs. (B) Comparison between the SRs of the jejunum. Evaluated data that passed the Shapiro–Wilk normality test were analyzed using the *t*‐test with Welch’s correction (^∗∗^ *p* < 0.01; ^∗∗∗^ *p* < 0.001).

In the SR of the animals in the EG, the values ranged from 1.01 to 3.3 in the duodenum, with a mean of 2.105 ± 1.016 and a 95% confidence interval of 1.039–3.171, and in the jejunum, the measurements ranged from 1.19 to 2.76, with a mean of 1.867 ± 0.5711 and a 95% confidence interval of 1.267–2.466. In the CG in the duodenum, the values ranged from 0.2 to 0.96, with a mean of 0.5667 ± 0.3133 and a 95% confidence interval of 0.2379–0.8954, and in the jejunum, the measurements ranged from 0.15 to 0.72, with a mean of 0.4667 ± 0.2439 and a 95% confidence interval of 0.2107–0.7227. Therefore, the animals in the EG had higher values in the elastography parameters compared to the CG. The difference in the SR values of the duodenum (*p* = 0.0053) and jejunum (*p* = 0.0003) between the groups was significant in the mean comparison tests (Figure 2).

**FIGURE 3:**
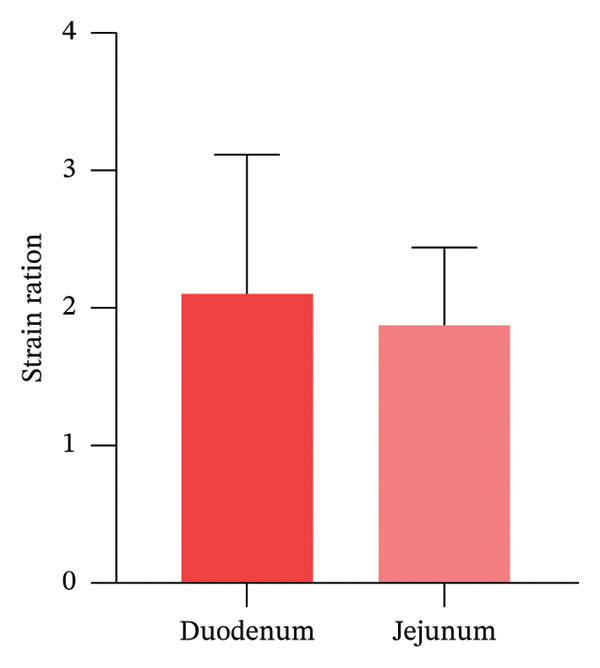
Mean values and standard deviation of the strain ratio (SR) of the duodenum and jejunum of dogs in the enteropathy group. Evaluated data that passed the Shapiro–Wilk normality test were analyzed using the *t*‐test with Welch’s correction (*p* = 0.6301).

In the EG animals, the SR averages were different between the intestinal segments evaluated. The values for the duodenum were 2.105 ± 1.016 and slightly higher when compared to the measurements for the jejunum 1.867 ± 0.5711. However, this difference was not statistically significant in the comparison test (*p* = 0.6301) (Figure 3).

**FIGURE 4:**
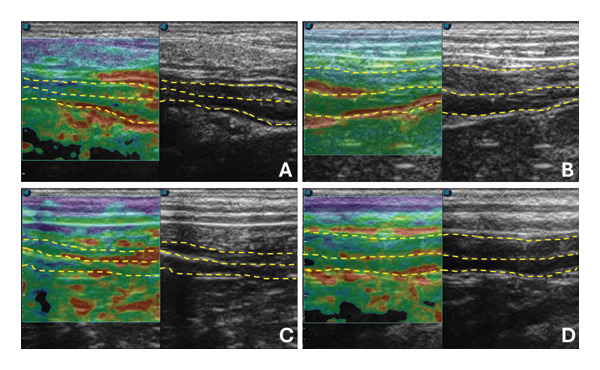
Color elastogram in double projection with the B‐mode ultrasound image of the longitudinal scan of the proximal jejunum. (A) Submucosa, muscularis, ventral and dorsal serosa red‐green, and ventral and dorsal mucosa blue‐green. (B) Submucosa, muscularis, and ventral serosa green; submucosa, muscularis, and dorsal serosa red; ventral mucosa blue‐red‐green; and dorsal mucosa green. (C) Mucosa, submucosa, muscle, and ventral serosa blue‐green; ventral mucosa red; submucosa, muscle, and dorsal serosa green‐blue‐red; and dorsal mucosa green‐blue. (D) Submucosa, muscularis, and ventral and dorsal serosa red‐green; dorsal and ventral mucosa green‐blue.

In addition to the semiquantitative measurements, a visual assessment was made of the color pattern among the animals in the EG. There were varied colorimetric matrices distributed throughout the intestinal mucosa. Figure 4 shows a compilation of the color histograms associated with the B‐mode USG image. To carry out this qualitative analysis, the image of the intestines was generated in a longitudinal section to promote better distribution of the elastogram filling in the mucosa. The jejunum was evaluated, where it was possible to observe heterogeneous dispositions between the ventral and dorsal portions of the intestinal segment with patterns ranging from predominantly green to blue and small reddish parts.

The clinical activity index for enteropathy in the EG dogs showed 33.3% (*n* = 2/6) with a score of 13, 16.6% with a score of 11 (*n* = 1/6), 16.6% with a score of 9 (*n* = 1/6), and 33.3% with a score of 6 (*n* = 2/6). Based on the reference literature [18], there were two dogs with very severe CIE, two with severe disease, and two with moderate degree. Spearman’s correlation was used to assess the relationship between the CCECAI scale, laboratory parameters, and intestinal elastography data in the EG. There was a weak correlation between intestinal SR and the variables assessed, except for a strong, negative, and statistically significant correlation (*r* = −0.886, *p* = 0.033) between jejunal SR and serum albumin (Table 2).

**TABLE 2:** Representation of the variables used to assess the correlation between duodenum and jejunum strain elastography data and laboratory and clinical parameters in the enteropathy group.

**Variables**		**SR duodenum**	**SR jejunum**	**Erythrocytes**	**PLT**	**Leukocytes**	**TPP**	**Albumin**	**CRP**	**Cholesterol**	**Cobalamin**	**Folate**	**CCECAI**

SR duodenum	*r*	1	−0.200	0.257	−0.657	−0.771	0.486	0.143	0.638	−0.429	−0.657	0.371	0.059
	*p* value	—	0.714	0.658	0.175	0.103	0.356	0.803	0.200	0.419	0.175	0.497	0.911

SR jejunum	*r*		1	0.257	−0.029	−0.371	−0.543	−0.886^∗^	−0.334	−0.029	0.543	0.314	0.294
	*p* value		—	0.658	1.00	0.497	0.297	0.033	0.533	1.00	0.297	0.564	0.544

Erythrocytes	*r*			1	−0.314	−0.371	0.086	−0.600	0.030	−0.714	−0.257	0.200	0.412
	*p* value			—	0.564	0.497	0.919	0.242	1.00	0.136	0.658	0.714	0.444

PLT	*r*				1	0.657	0.257	0.029	0.152	0.829	0.314	−0.029	−0.029
	*p* value				—	0.175	0.658	1.00	0.833	0.058	0.564	1.00	0.978

Leukocytes	*r*					1	−0.143	0.429	−0.273	0.486	0.086	−0.714	0.088
	*p* value					—	0.803	0.419	0.617	0.356	0.919	0.136	0.867

TPP	*r*						1	0.314	0.880^∗^	0.200	−0.486	0.429	−0.177
	*p* value						—	0.564	0.050	0.714	0.356	0.419	0.774

Albumin	*r*							1	0.273	0.257	−0.486	−0.486	−0.206
	*p* value							—	0.617	0.658	0.356	0.356	0.700

CRP	*r*								1	0.273	−0.638	0.334	0.156
	*p* value								—	0.617	0.200	0.533	0.800

Cholesterol	*r*									1	0.257	−0.029	−0.088
	*p* value									—	0.658	1.00	0.867

Cobalamin	*r*										1	0.314	−0.500
	*p* value										—	0.564	0.311

Folate	*r*											1	−0.471
	*p* value											—	0.356

CCECAI	*r*												1
	*p* value												—

*Note:* The data passed the Shapiro–Wilk normality test and were analyzed using Spearman’s correlation.

^∗^Significant at 5% probability level.

On the USG of the EG animals, the mean values for measuring the duodenum were 0.4867 ± 0.07554 cm and those for the jejunum were 0.3850 ± 0.1089 cm. Spearman’s correlation test was used to analyze the relationship between the SR of the duodenum and jejunum and the measurement of the same intestinal layers by USG, as well as the evaluation between the variables themselves. Thus, we observed a weak correlation between the elastography measurements and the variables evaluated, except for a strong positive relationship between the USG measurement of the duodenum and jejunum in centimeters (*r* = 0.82), but without statistical significance (*p* = 0.067).

In the evaluation carried out by upper digestive endoscopy to analyze the duodenum, prior to the biopsy, a visual assessment was made of the morphological aspects of the duodenal mucosa of the EG animals. The criteria evaluated were hyperemia, edema, friability, hemorrhage, erosion/ulceration, and lacteal dilation. The scores were established individually between the animals (Table 3).

**TABLE 3:** Endoscopic parameters for assessing the severity of lesions detectable by duodenoscopy in dogs with chronic inflammatory enteropathy.

Dogs	Hyperemia	Edema	Friability	Hemorrhage	Erosion/ulceration	Lacteal dilation
1	1	1	1	0	0	0
2	2	2	1	0	0	1
3	3	3	2	0	0	2
4	2	3	2	0	0	2
5	2	3	2	0	0	2
6	2	2	2	0	0	2

*Note:* (0) normal mucosa or no detectable lesions, (1) mild lesions, (2) moderate lesions, and (3) severe lesions.

**FIGURE 5:**
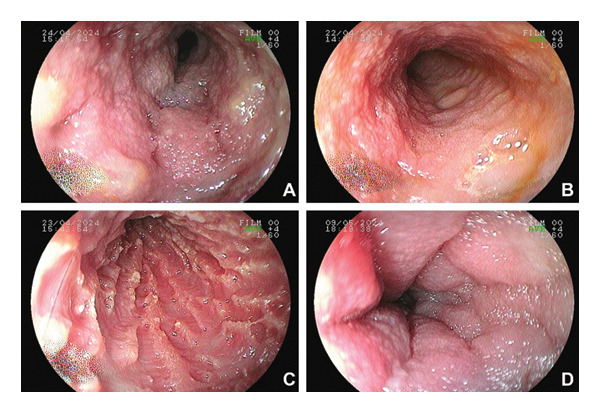
Duodenoscopy of dogs in the enteropathy group. (A) Increased granulosity of the duodenal mucosa associated with the presence of whitish elevations and surface irregularity. (B) Presence of bile secretion and granularity of the mucosa. (C) Lacteal dilatation with a raised, whitish appearance on the duodenal mucosa. (D) Enanthema of the mucosa associated with granulosity.

The changes seen in the duodenum during endoscopy are shown in Figure 5. The endoscopic scores were correlated with the hardness rates in the EG animals. For this reason, the values of the variables were submitted to the Spearman test. The endoscopic parameters hemorrhage and erosion/ulceration were not considered in the statistical test since they were not detected in any animal in the group. Thus, there was a weak correlation between duodenal SR and all the duodenoscopy data, ranging from *r* = −0.088 to 0.169, with no statistical significance.

The samples collected by endoscopic biopsy were evaluated separately in terms of the lesions found. Fifteen duodenum samples were taken from each animal in the EG, giving a total of 90 samples. Of these, 23.3% (*n* = 21/90) were inadequate, 39% (*n* = 35/90) were marginal, and 37.7% (*n* = 34/90) were adequate.

The histopathological severity indices were correlated with the hardness rates in the EG animals. For this reason, the sample values were applied to Spearman’s correction test. The histological findings of shortened villi, mucosal fibrosis, eosinophils, neutrophils, and macrophages in the lamina propria were not considered in the statistical test as they were not detected in any of the animals in the group. In view of the above, there was a weak correlation between the elasticity of the duodenum and histological criteria, which ranged from *r* = −0.655 to 0.507 with no statistical significance.

## 4. Discussion

The results obtained indicate that elastography is feasible and may be useful for identifying inflamed intestinal mucosa compared with normal intestinal mucosa. These findings are partially consistent with those reported by a previous study [6], which found the test to be useful in assessing the healthy mucosa of the jejunum of dogs. Despite the differences in methodology, the recommendation to use the technique served as the basis for this pioneering study in dogs with CIE. Several deformation elastography studies have been carried out in people with inflammatory bowel disease (IBD) [22–25].

However, although they share our objective of detecting patterns suggestive of intestinal fibrosis, these data from the literature still focus on the early detection of stenosis, a development that the authors cite as frequent in people with IBD, but uncommon in dogs with a similar disease, CIE [2]. Since there are no directly comparable veterinary studies, we suggest that the particularities of human and canine intestinal diseases should be considered in elastography applications.

The association between the CCECAI of the EG dogs and the values obtained from elastography was evaluated, and a weak correlation was found with duodenal and jejunal stiffness. One study obtained similar results when correlating the Harvey–Bradshaw index, which aims to identify the state of inflammatory activity in people with Crohn’s disease, with the SR [25]. In contrast, the same index was positively correlated with acoustic radiation force impulse (ARFI) evaluation of the intestines in humans [26]. In view of this, we believe that elastography findings may not correspond directly with clinical symptoms and that prospective studies are needed to compare intestinal elasticity with clinical signs in dogs with different chronic enteropathies.

Previous studies, despite comparing different variables, have obtained results that contrast with the present study, by verifying correspondence between the clinical index and digestive endoscopy, and histopathology of the duodenum [21, 27] and colon [21]. In addition, one study found a significant relationship between the USG measurement and a clinical scale [28], while other authors have demonstrated a strong relationship between contrast‐enhanced US parameters and the CCECAI [29]. The lack of correlation seen in this study does not support the idea of intestinal elasticity being a marker for the clinical follow‐up of enteropathies, but further analysis is needed with a larger sample of dogs and a prospective comparison of the variables.

Spearman’s test between the rates of intestinal hardness and the laboratory parameters erythrocytes, total leukocytes, TPP, platelets, and total cholesterol in the sick dogs showed a weak correlation and no statistical significance. We therefore believe that, in this study, the hematological and biochemical markers did not change with the increased rigidity of the intestinal mucosa in dogs with enteropathy. These findings are similar to those reported in medical studies [25, 30], which found no association between laboratory data and strain elastography. However, both studies cited correlated with fecal calprotectin, which was not included in this experiment. We conclude that the use of inflammatory, absorption, and microbiological biomarkers should be compared with intestinal stiffness in future studies, given their less nonspecific nature for assessing gastrointestinal function and injury.

When evaluating the relationship between CRP and intestinal elasticity parameters, a moderate, positive, and statistically insignificant correlation was found with duodenal SR and a weak, negative, and statistically insignificant correlation was found with jejunal SR in the EG. These results are similar to those of a medical study, in which 26 people with symptomatic stenosing bowel disease were assessed and the tension ratio did not correlate with CRP [25]. Despite the similarities in methodology and objectives, the authors cited in Ref. [25] carried out a prospective evaluation prior to surgery and the elastographic data were obtained from stenosed segments. In contrast, the present study involved a single evaluation and compared the findings with those of the CG, assessing the intestinal mucosa in dogs without luminal narrowing.

As for the absorption markers carried out in the group with enteropathy, no statistically significant associations were found between the serum value of cobalamin and duodenal SR, cobalamin and jejunal SR, folate and duodenal SR, and folate with jejunal SR. In the only experimental study comparable to ours, the dosages of these vitamins were carried out in the dogs, but the correlation tests were not carried out [6]. We believe that the results may have been influenced by the fact that cobalamin is absorbed in the ileum [31] and this intestinal segment was not evaluated in this study. Furthermore, although folic acid is absorbed mainly in the jejunum [32], it is important to consider that elastography was only carried out in the proximal region, so a complete mapping of this intestinal segment could better define the relationship between jejunal elasticity and the serum concentration of vitamin B9. Finally, it is known that these biomarkers are influenced by intestinal dysbiosis [33]. We recognize the importance of new analyses with the inclusion of intestinal microbiological markers such as the dysbiosis index.

A strong, negative, and statistically significant correlation was observed between jejunal SR and serum albumin. Thus, the hardness of the jejunum was inversely associated with the concentration of albumin, and we therefore consider that acute inflammation increases the hardness of the mucosa and consequently can promote malabsorption. This corroborates the authors who mention albumin as a parameter for assessing intestinal absorption [18]. However, for this interpretation, it is important to consider other markers, such as the serum and fecal alpha 1 proteinase inhibitory factor, which has early potential in verifying intestinal protein loss [34]. In addition, other diseases that promote hypoproteinemia should be considered, such as glomerulopathies, liver failure, malnutrition, and acute inflammation [35]. In this study, by means of the inclusion criteria and the screening that the EG dogs underwent, we were able to exclude extraintestinal causes of hypoalbuminemia. Despite this, we would highlight the importance of performing a urinary protein–creatinine ratio to diagnose renal protein loss, a test that was not carried out on the animals in this study. Finally, the relationship between intestinal mucosal hardness and malabsorption is still uncertain. For further clarification, we suggest experimental studies with dogs that specifically present protein‐losing enteropathy.

The results show that there was a discrepancy in the SR of the duodenum and jejunum when comparing the means and standard deviation between the EG and CG. The difference in the values of the duodenum (*p* = 0.0053) and jejunum (*p* = 0.0003) between the groups was statistically significant. This corroborates partially published data in which intestinal hardness rates were higher in dogs with enteropathy compared to data obtained from the jejunum of healthy dogs [6]. Several human studies have obtained similar findings to this experiment, in which strain elastography confirmed greater intestinal hardness in individuals with enteropathy [23–25, 36].

A recent update on canine chronic enteropathy does not show whether there are intestinal segments that are more affected by the inflammatory process in the different classifications of the disease [2]. Based on this, in our initial hypothesis, we did not believe in differences in inflammation and consequently discrepancies in elasticity rates between the intestines of sick animals. According to the authors in Ref. [2] and our hypothesis, in the EG animals, the semiquantitative values of the deformation rates were not statistically significant. Despite this, there was a disparity in the values, with 2.105 ± 1.016 in the duodenum and 1.867 ± 0.5711 in the jejunum. In contrast to medicine, in which the ileum is the intestinal segment most affected by Crohn’s disease, some authors choose this part of the small intestine for elastographic study [25, 37, 38].

Fufezan et al. [36] proposed a colorimetric scoring system for assessing the activity of Crohn’s disease in pediatrics. Based on this, they suggested inflammation and fibrosis of the intestinal wall by means of the color map in three types: intestine in remission (blue/green/blue), inflamed mucosa (green/blue), and fibrotic mucosa (blue without color stratification). Based on this scale, we can extrapolate that the EG animals would fall into the classification of inflamed mucosa, since the qualitative analysis revealed colorimetric patterns that varied mostly from green to blue and small red parts. However, this system was not correlated with histopathology [36], which represents a major weakness in its application. Furthermore, it is important to consider that there are differences between the etiopathogenesis of canine and human intestinal inflammation. We believe that visual observation in elastography is a quick and noninvasive method, but interpretation may be challenging if there are no specific classification systems for dogs that can be repeated and reproduced in subsequent studies.

The mean USG values of the duodenum and jejunum were 0.4867 ± 0.07554 cm and 0.3850 ± 0.1089 cm, respectively. When evaluating the correlation between USG and SR, there were no strong, statistically relevant correlations. However, between the same variables, there was only one strong correlation without statistical significance (*r* = 0.82, *p* = 0.067) between the measurements of the intestinal segments studied. This agrees with the description by Giannetti et al. [39] who determined that B‐mode imaging shows an increase in the thickness of the intestinal wall in people with enteropathies. Despite this, our results disagree with those of the same authors [39] who found a correlation between USG intestinal thickening and increased semiquantitative rates on elastography. We suggest that in future studies, in addition to increasing the sample of animals, a correlation should be made between SR and total wall thickness and specifically with the diameter of the mucosal layer.

In the upper digestive endoscopy to evaluate the duodenum of the sick animals, a weak correlation was observed with duodenal SR with all the duodenoscopy data, which ranged from *r* = −0.088 to 0.169 without statistical significance. This differs from the medical study that found an association between intestinal mucosal edema in people with Crohn’s disease and an increased SR [39]. Salavati et al. [40] found a correlation between the endoscopic score and contrast‐enhanced USG in the duodenum of dogs with CIE; however, despite the study using minimally invasive diagnostic imaging techniques, elastography was not included in the methodology.

Several medical studies have aimed to evaluate the potential of elastography in the early detection of intestinal fibrosis and the application of this test for the differential diagnosis of inflammation of fibrotic tissue [24, 25, 39–45]. Like the authors, we shared the same objective in this study. However, a weak correlation was observed between the elasticity of the duodenum and histopathological criteria, ranging from *r* = −0.655 to 0.507, with no statistical significance. Furthermore, it was not possible to perform a correlation test between duodenal SR and mucosal fibrosis, since this alteration was not found in any of the samples in the scoring system. As such, we were unable to define exactly whether we were corroborating the authors who found no association between fibrosis and deformation elastography [25] or those who obtained statistically significant relationships [24, 39, 41, 42].

We recognize as a limitation of this study the nonuse of histological staining that is more suitable for detecting fibrotic tissue, as performed by Zidar et al. [43], who used Massom’s trichrome in the samples of the study that aimed to better understand intestinal fibrosis. However, the authors of Ref. [21] who standardized the histological scoring performed on the EG animals do not specify the use of histopathology stains. The number of endoscopic biopsy samples from the duodenum exceeded the number recommended by worldwide recommendations [20], but many of the fragments analyzed were not considered in the statistical tests because they were marginal or inadequate. We believe that tissue collection may have been compromised by intestinal inflammation, as reported in the literature [44].

## 5. Conclusion

Based on the results obtained, we conclude that strain elastography can be applied to dogs with CIE, offering an additional tool for assessing the intestinal mucosa. The research showed a weak correlation between intestinal stiffness and clinical and laboratory parameters, indicating that mucosal elasticity may not directly reflect the clinical severity of the disease. Elastography did not allow for differentiation between inflammation and intestinal fibrosis in the present study, but it did allow for the identification of increased mucosal rigidity in animals with enteropathy. Although the technique proved to be safe and applicable, we highlight the need for future studies with larger samples and more comprehensive comparative methods to validate and refine its diagnostic use, such as the use of shear wave elastography and more sensitive markers for intestinal fibrosis.

**Nomenclature**
ALP: Alkaline phosphatase
ALT: Alanine aminotransferase
ARFI: Acoustic Radiation Force Impulse
CCECAI: Canine Chronic Enteropathy Clinical Activity Index
CG: Control group
CI: Confidence interval
CIE: Chronic inflammatory enteropathy
CRP: C‐reactive protein
EDTA: Ethylenediaminetetraacetic acid
EG: Enteropathy group
IBD: Inflammatory bowel disease
MHz: Megahertz
PLT: Platelets
ROI: Region of interest
SR: Strain ratio
TPP: Total plasma proteins
USG: Ultrasound

## Author Contributions

Iago Martins Oliveira, Naida Cristina Borges, Wanessa Patrícia Rodrigues Da Silva, Rafaela Rodrigues Ribeiro, Maria Eduarda Cardoso Cysneiros, Vanessa Rezende Moraes, Lucas Rodrigues Ferreira, Marco Augusto Machado Silva, and Rafael Antônio Lopes Xavier conceptualized the study. Iago Martins Oliveira and Naida Cristina Borges developed the methodology. Iago Martins Oliveira and Wanessa Patrícia Rodrigues Da Silva performed the formal analysis. Iago Martins Oliveira, Rafaela Rodrigues Ribeiro, and Naida Cristina Borges analyzed the data. Iago Martins Oliveira wrote the original draft. Maria Eduarda Cardoso Cysneiros, Vanessa Rezende Moraes, Lucas Rodrigues Ferreira, Murilo Rodrigues De Souza, and Rafael Antônio Lopes Xavier reviewed and edited the manuscript.

## Funding

No funding was received for this research.

## Disclosure

All authors have read and approved the final version of the manuscript. The corresponding author had full access to all the data in this study and takes complete responsibility for the integrity of the data and the accuracy of the data analysis.

## Ethics Statement

The study was approved by the Animal Use Ethics Committee (CEUA) of the Federal University of Goiás (UFG), under protocol numbers 71/20 and 41/24.

## Conflicts of Interest

The authors declare no conflicts of interest.

## Data Availability

The data that support the findings of this study are available from the corresponding author upon reasonable request.
